# Impact of Plasma Xanthine Oxidoreductase Activity on the Mechanisms of Distal Symmetric Polyneuropathy Development in Patients with Type 2 Diabetes

**DOI:** 10.3390/biomedicines9081052

**Published:** 2021-08-19

**Authors:** Midori Fujishiro, Hisamitsu Ishihara, Katsuhiko Ogawa, Takayo Murase, Takashi Nakamura, Kentaro Watanabe, Hideyuki Sakoda, Hiraku Ono, Takeshi Yamamotoya, Yusuke Nakatsu, Tomoichiro Asano, Akifumi Kushiyama

**Affiliations:** 1 Division of Diabetes and Metabolic Diseases, Department of Internal Medicine, Nihon University School of Medicine, 30-1 Oyaguchi Kami-cho, Itabashi-ku, Tokyo 173-8610, Japan; ishihara.hisamitsu@nihon-u.ac.jp (H.I.); watanabe.kentaro@nihon-u.ac.jp (K.W.); 2 Department of Internal Medicine, Nihon University Hospital, 1-6 Kanda-Surugadai, Chiyoda-ku, Tokyo 101-8309, Japan; ogawa.katsuhiko@nihon-u.ac.jp; 3 Division of Neurology, Department of Internal Medicine, Nihon University School of Medicine, 30-1 Oyaguchi Kami-cho, Itabashi-ku, Tokyo 173-8610, Japan; 4 Radioisotope and Chemical Analysis Center, Pharmaceuticals Research Laboratories, Sanwa Kagaku Kenkyusho Co., Ltd., 363 Shiosaki, Hokusei-cho, Inabe-shi 511-0406, Mie, Japan; ta_murase@skk-net.com; 5 Medical Affairs Department, Sanwa Kagaku Kenkyusho Co., Ltd., 35 Higashisotobori-cho, Higashi-ku, Nagoya 461-8631, Aichi, Japan; ta_nakamura@mb4.skk-net.com; 6 Division of Neurology, Respirology, Endocrinology and Metabolism, Department of Internal Medicine, Faculty of Medicine, University of Miyazaki, 5200 Kihara, Kiyotake 889-1692, Miyazaki, Japan; hideyuki_sakoda@med.miyazaki-u.ac.jp; 7 Department of Clinical Cell Biology, Graduate School of Medicine, Chiba University, 1-8-1 Inohana, Chuo-ku, Chiba 260-8670, Chiba, Japan; hono@chiba-u.jp; 8 Department of Medical Science, Graduate School of Medicine, Hiroshima University, 1-2-3 Kasumi, Minami-ku, Hiroshima 734-8551, Hiroshima, Japan; ymmty@hiroshima-u.ac.jp (T.Y.); nakatsu@hiroshima-u.ac.jp (Y.N.); tasano@hiroshima-u.ac.jp (T.A.); 9 Department of Pharmacotherapy, Meiji Pharmaceutical University, 2-522-1 Noshio, Kiyose 204-8588, Tokyo, Japan; kushiyama@my-pharm.ac.jp

**Keywords:** type 2 diabetes, xanthine oxidoreductase, distal symmetric polyneuropathy

## Abstract

To unravel associations between plasma xanthine oxidoreductase (XOR) and diabetic vascular complications, especially distal symmetric polyneuropathy (DSP), we investigated plasma XOR activities using a novel assay. Patients with type 2 diabetes mellitus (T2DM) with available nerve conduction study (NCS) data were analyzed. None were currently taking XOR inhibitors. XOR activity of fasting blood samples was assayed using a stable isotope-labeled substrate and LC-TQMS. JMP Clinical version 5.0. was used for analysis. We analyzed 54 patients. Mean age was 64.7 years, mean body mass index was 26.0 kg/m^2^, and mean glycated hemoglobin was 9.4%. The logarithmically transformed plasma XOR activity (ln-XOR) correlated positively with hypoxanthine, xanthine, visceral fatty area, and liver dysfunction but negatively with HDL cholesterol. ln-XOR correlated negatively with diabetes duration and maximum intima-media thickness. Stepwise multiple regression analysis revealed ln-XOR to be among selected explanatory factors for various NCS parameters. Receiver operating characteristic curves showed the discriminatory power of ln-XOR. Principal component analysis revealed a negative relationship of ln-XOR with F-waves as well as positive relationships of ln-XOR with hepatic steatosis and obesity-related disorders. Taken together, our results show plasma XOR activity to be among potential disease status predictors in T2DM patients. Plasma XOR activity measurements might reliably detect pre-symptomatic DSP.

## 1. Introduction

Type 2 diabetes mellitus (T2DM) is a metabolic disease that leads to various vascular complications involving multiple organs, ultimately reducing the lifespans of affected patients [1]. Diabetes prevalence rose in the second decade of the 21st century and continues to increase. In 2019, diabetes prevalence worldwide was estimated to be 463 million people [2], and 4.2 million people were estimated to have died from diabetes and its complications [3]. Prevention or early detection of T2DM and its complications is hugely important but occult and asymptomatic complications are difficult to detect. Distal symmetric polyneuropathy (DSP) especially often precedes other complications and can progress to become a life-threatening disorder [4]. However, the progression of DSP is very difficult to evaluate and employs routine outpatient examinations. Consensus definitions consistently recommend a combination of neuropathic symptoms and signs in addition to specific nerve conduction study (NCS) abnormalities as criteria for diagnosing DSP and it is essential to confirm that the diagnosis of this condition is accurate [5].

Though the mechanisms by which diabetic vascular complications develop remain to be elucidated, oxidative stress and chronic inflammation, as well as longstanding hyperglycemia, associated metabolic derangements including increased polyol flux, accumulation of advanced glycation end products, and lipid alterations among other metabolic abnormalities, are thought to be among the key factors contributing to the development of DSP [6,7]. Obesity, especially when accompanied by visceral fat accumulation, hypertension, hyperglycemia, hyperinsulinemia, and fatty change in the liver are considered to be parameters predicting upregulation of oxidative stress and/or chronic inflammation as risk factors for diabetic complications [8]. The serum uric acid (UA) level is also associated with obesity and insulin resistance [9,10], and a high serum UA level has been proposed to be an independent risk factor for various diabetic complications such as diabetic nephropathy [11,12], as well as diabetic retinopathy (DR) [13] and neuropathy [14]. Xanthine oxidoreductase (XOR), the rate-limiting enzyme of UA production, was recently reported to be upregulated in fat, liver, kidneys, and the vasculature of patients with T2DM and other metabolic diseases [15,16]. XOR produces UA by catalyzing the oxidation of purines such as hypoxanthine to xanthine and xanthine to UA. Excessive purine derivatives derived from biological activities such as ATP depletion due to exercise, intake of fructose or alcohol, intake of a purine-rich diet or a pathological event such as ischemia, or degradation of RNA and DNA induced by cell turnover, are broken down by the purine metabolism system, in which XOR plays an essential role [17]. On the other hand, xanthine dehydrogenase (XDH) is converted to xanthine oxidase (XO) in response to tissue injury [18]. XO can reduce molecular oxygen to superoxide and hydrogen peroxide and is thought to be one of the key enzymes producing reactive oxygen species (ROS) [19] which serve as important messengers inducing inflammation [20,21].

However, XOR activity measurement has not been sufficiently established because the level of plasma XOR activity is quite low in humans as compared with that in rodents [17]. Recently, a novel human plasma XOR activity assay using a combination of liquid chromatography (LC) and triple quadrupole mass spectrometry (TQMS) to detect [13C2,15N2]-UA using [13C2,15N2]-xanthine as a substrate was developed [22]. At present, plasma XOR activity levels during the clinical courses of T2DM patients remain unclear. Thus, to unravel the associations of early vascular complications such as DSP with XOR activity, we investigated the relationships between clinical features of T2DM and plasma XOR levels measured employing this novel assay.

## 2. Materials and Methods

### 2.1. Study Subjects

We enrolled 127 patients with T2DM who visited the department of diabetes and metabolic diseases in our hospital during the period from August 2017 to October 2020. Of these patients, we analyzed those who had complete NCS data. The key inclusion criteria were as follows: (1) confirmed diagnosis of T2DM, (2) 18 years or older, and (3) no liver dysfunction. The exclusion criteria were: (4) pregnant, breastfeeding, or not using contraception for women of childbearing age, (5) currently taking XOR inhibitors, and/or (6) judged by their primary doctors to be inappropriate for trial enrollment due to safety concerns or for any other reasons. This investigation conformed with the principles outlined in the Declaration of Helsinki. The study protocol was approved by the Institutional Ethics Committee of Nihon University Hospital (approval number 20170701) and was registered at UMIN Clinical Trials Registry with the ID number UMIN000029257. All patients provided written informed consent for study participation.

### 2.2. Clinical Parameters and Procedures

Patient profiles, including diabetic microangiopathy, were collected from medical records. The intra-abdominal visceral fat area (VFA) was evaluated from computed tomography cross-sectional scans at the level of the umbilicus, as previously reported [23]. Diabetic nephropathy was clinically diagnosed by attending physicians based on microalbuminuria or overt proteinuria with no evidence of other kidney or urological diseases [24] and was classified into four stages (patients receiving dialysis therapy were excluded) according to the classification of diabetic nephropathy promulgated in 2014 by a joint committee on diabetic nephropathy in Japan [25]. DR includes non-proliferative DR diagnosed by the presence of microaneurysms and retinal hemorrhages [26], and all DR cases were confirmed by ophthalmologists. DR was subdivided as follows: no apparent diabetic retinopathy (NDR), simple diabetic retinopathy (SDR), and proliferative diabetic retinopathy (PDR). For the estimation of diabetic autonomic neuropathy, the coefficient of variation of the R-R intervals at rest and in deep breathing was measured with CardiMax FCP-8800^®^ (Fukuda Denshi Corporation, Tokyo, Japan) as previously described [27]. To estimate DSP, the vibratory sensation was tested by measuring the perception time for a fork vibration using a 128 Hz tuning fork at the lateral malleoli. We measured the sensory nerve conduction velocity of the sural nerve as well as motor nerve conduction velocity and F-wave parameters of the peroneal nerve and tibial nerves using NeuropackX1^®^ (Nihon Kohden Corporation, Tokyo, Japan) as previously described [27]. For estimation of macrovascular complications, we measured the cardio-ankle vascular index as well as the ankle-brachial index (ABI) using the VaSera VS-3000TN^®^ (Fukuda Denshi Corporation, Tokyo, Japan). We measured carotid ultrasonographic variables such as the intima-media thickness (IMT) and the plaque score of the common carotid artery as previously reported [28] using the Aplio 500^®^ (Toshiba Medical Systems Corporation, Tokyo, Japan). We usually performed the examinations, other than NCS, employed in this protocol as part of a comprehensive annual check-up aimed at detecting complications in patients with T2DM followed at our hospital.

To measure XOR activity, fasted blood samples were centrifuged at 3000 g for 15 min at 4 °C when collected in the early morning and the obtained plasma was stored at −80 °C until analysis. The XOR activity assay was performed using a stable isotope-labeled substrate and LC-TQMS together with the measurement of metabolites as previously reported [22,29]. In brief, 100 μL plasma samples pooled at −80 °C (purified by Sephadex G25 column) were mixed with Tris buffer (pH 8.5) containing [13C2,15N2]-xanthine as a substrate, NAD+, and [13C3,15N3]-UA as the internal standard. The mixtures were incubated at 37 °C for 90 min. Subsequently, the mixtures were combined with 500 μL of methanol and centrifuged at 2000× *g* for 15 min at 4 °C. The supernatants were transferred to new tubes and dried with a centrifugal evaporator. The residues were reconstituted with 150 μL of distilled water, filtered through an ultrafiltration membrane, and measured using LC/TQMS. We used LC/TQMS consisting of a Nano Space SI-2 LC system (Shiseido, Ltd., Tokyo, Japan) and a TSQ-TQMS (Thermo Fisher Scientific, Bremen, Germany) equipped with an ESI interface. Calibration standard samples of [13C2,15N2]-UA were also measured, and the amounts of [13C2,15N2]-UA production were quantitated from the calibration curve. XOR activities were expressed as [13C2,15N2]-UA in pmol/mL/h.

### 2.3. Statistical Analysis

JMP Clinical version 5.0 (SAS Institute, Cary, NC, USA) was used for all statistical analyses. Data are presented as the mean ± standard deviation, or as a number with the percentage. F-wave latency was corrected by height as previously described, that is, it was multiplied by 160 then divided by height (cm) [30]. We used Student’s *t*-test for continuous variables. Percentage data were examined using the Chi-square test or Fisher’s exact test as appropriate. Stepwise multiple regression analysis was performed using various NCV parameters stratified by the thresholds identified for prediction of incident DSP as described in a previous report [31] as the objective variable, as well as variables including XOR activity which had been natural logarithm-transformed to normalize the skewed distribution, sex, age, height, duration of diabetes, body mass index (BMI), waist circumference, fasting blood glucose (FBG), and maximum IMT as explanatory variables. To reveal the relationships between plasma XOR, DSP parameters, and other parameters in detail, we performed principal component analysis (PCA). When NCS values were undetectable, the longest F-wave latency in the dataset from another subject as well as a value of “0” for amplitude or conduction velocity was used as a substitute value for the purposes of the regression analysis as previously reported [32,33]. All *p*-values are two-sided, and *p* < 0.05 was considered to indicate a statistically significant difference.

## 3. Results

### 3.1. Characteristics of the Enrolled Patients

The characteristics and principal parameters of enrolled patients are listed in Table 1. We enrolled 54 patients with T2DM (37 males and 17 females, mean age 64.7 ± 12.2 years). Mean diabetes duration was 146 ± 130 months, glycated hemoglobin 9.4 ± 1.9%, BMI 26.0 ± 5.9 kg/m^2^, and waist circumference 94.1 ± 14.8 cm. As for antidiabetic drugs, 13 patients (24%) were prescribed various forms of insulin, 9 (17%) sulfonylureas or glinides, 27 (50%) dipeptidyl peptidase-4 inhibitors or GLP-1 agonists, 12 (22%) sodium-glucose cotransporter 2 inhibitors, 15 (28%) biguanides, 2 (4%) thiazolidinediones, 5 (9%) α-glucosidase inhibitors, and 1 (2%) epalrestat, as indicated in Table 1. As for antihypertensive agents, 17 patients (31%) were taking angiotensin-converting enzyme inhibitors/angiotensin receptor blockers, 15 (28%) calcium channel blockers, 3 (6%) diuretics, and 6 (11%) β-blockers.

### 3.2. Relationships of Plasma XOR Activity with Individual Parameters

**Figure 1:**
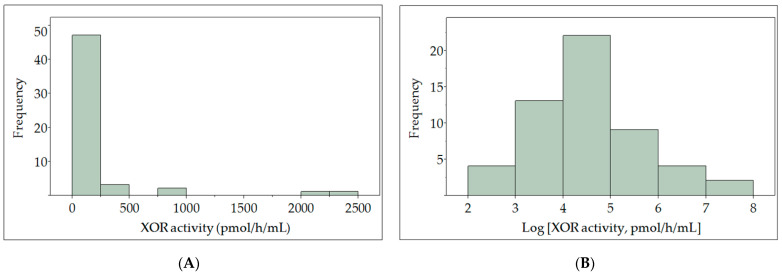
Distribution of plasma XOR activity (**A**) with logarithmically transformed data (**B**) displayed as a histogram.

**Figure 2:**
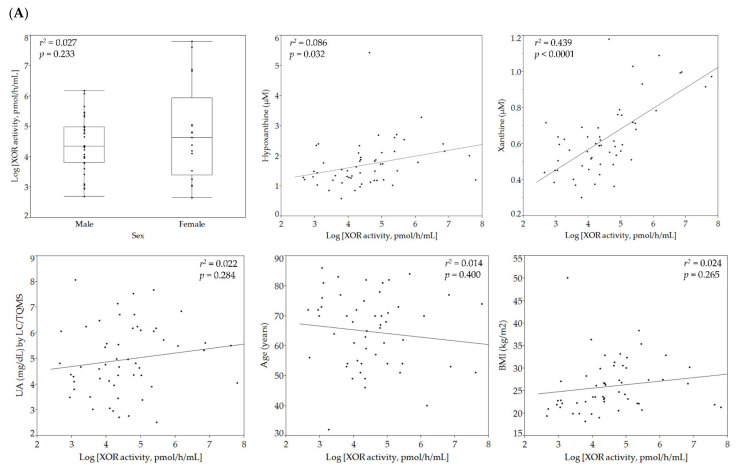

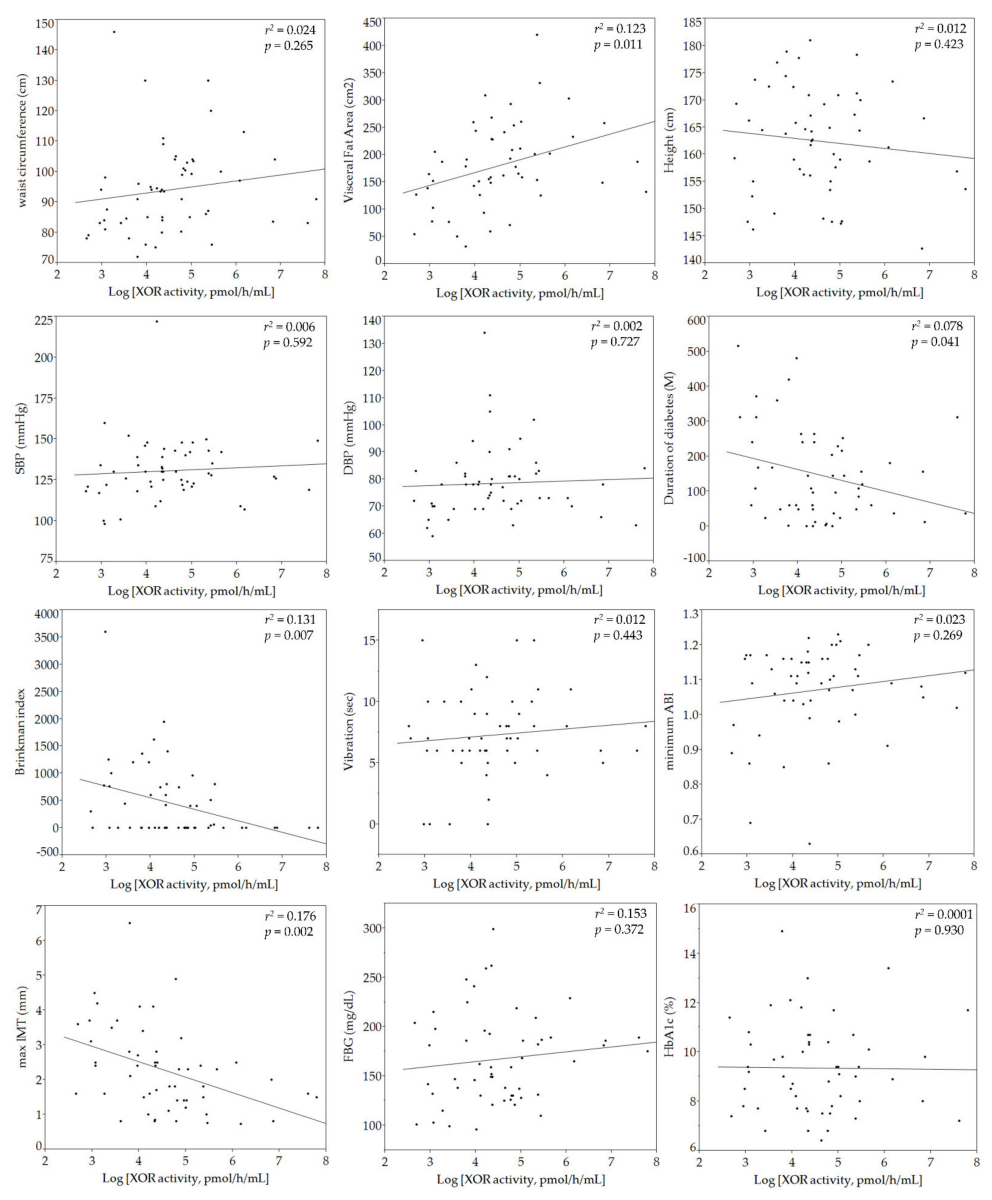

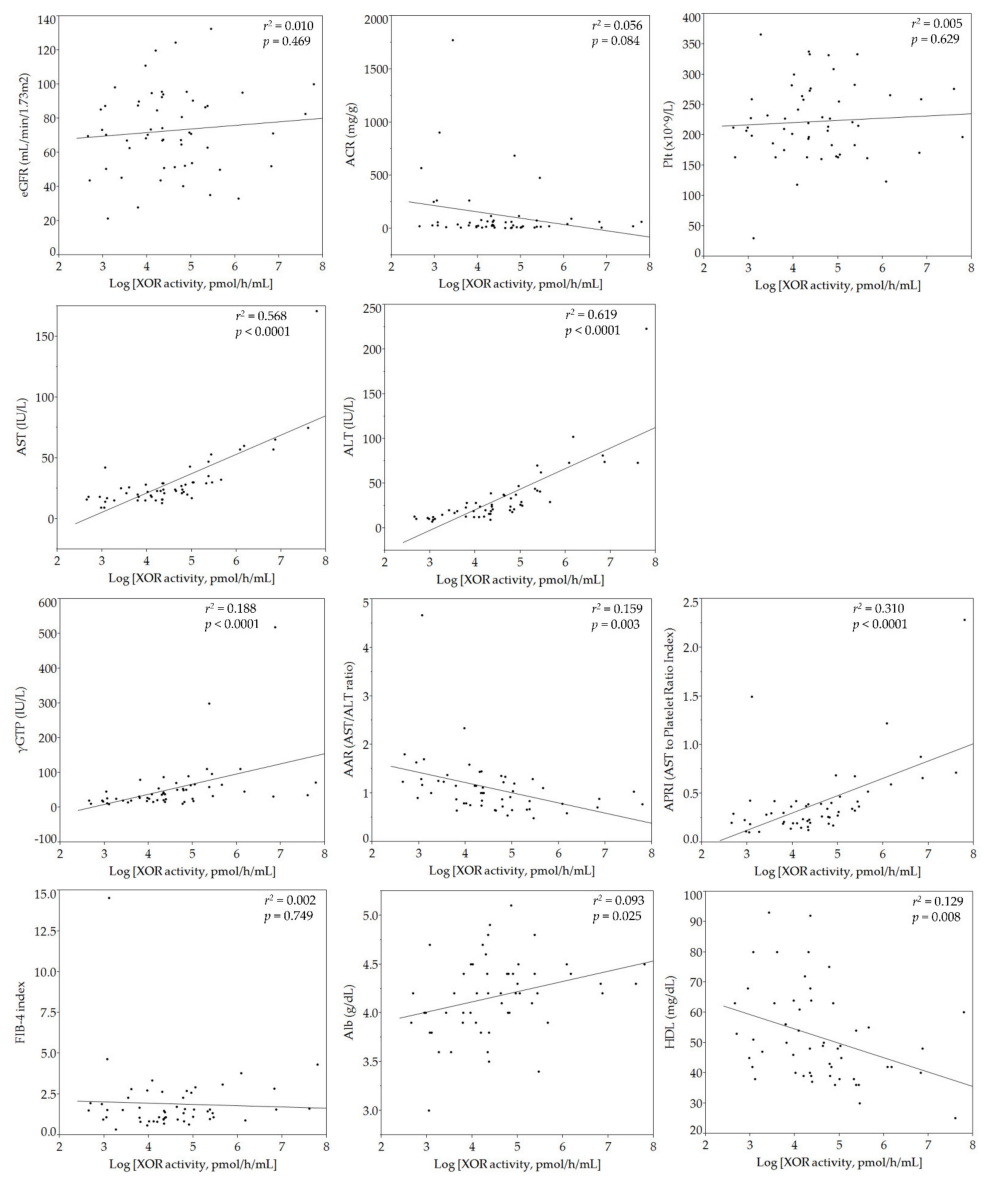

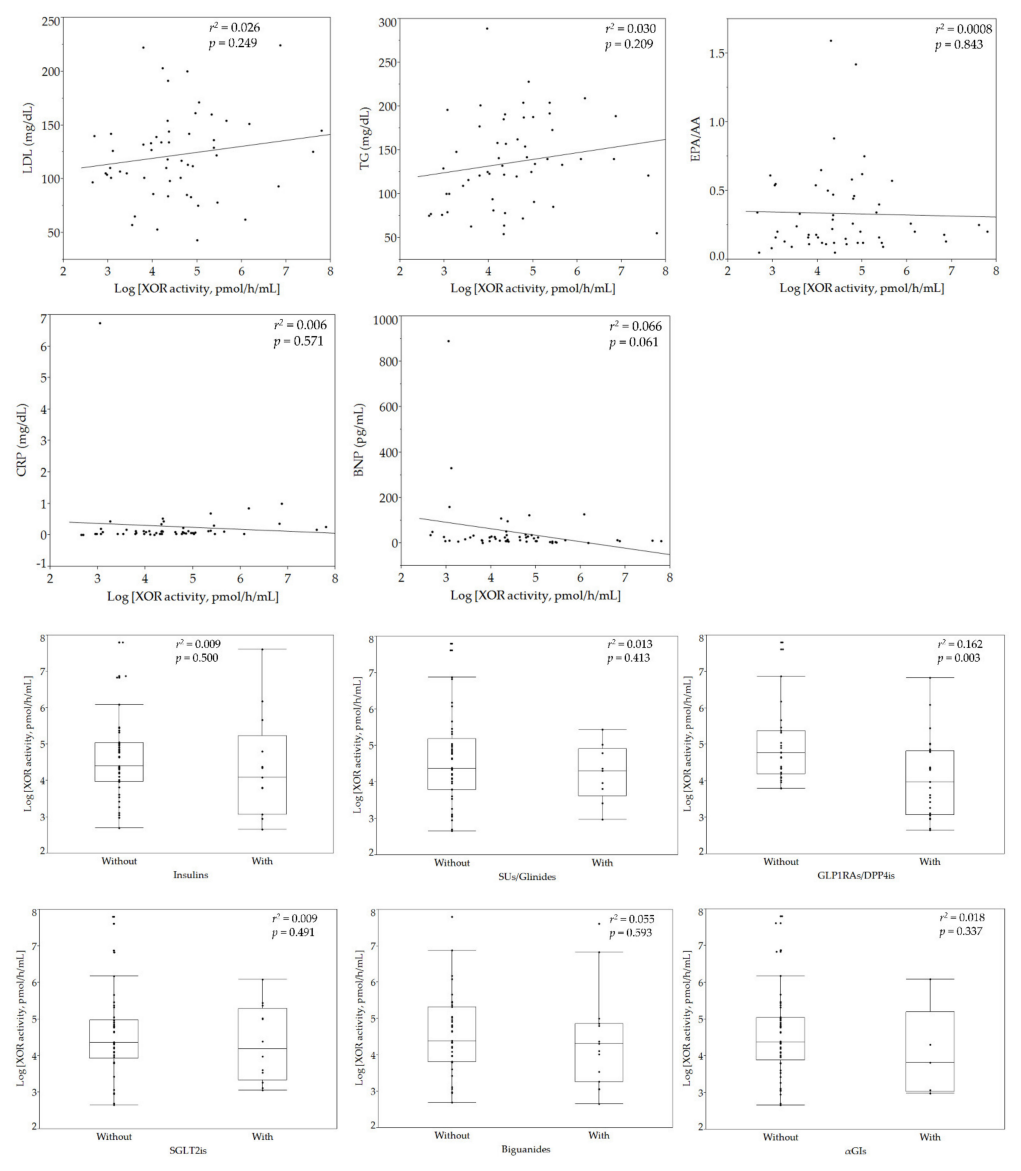

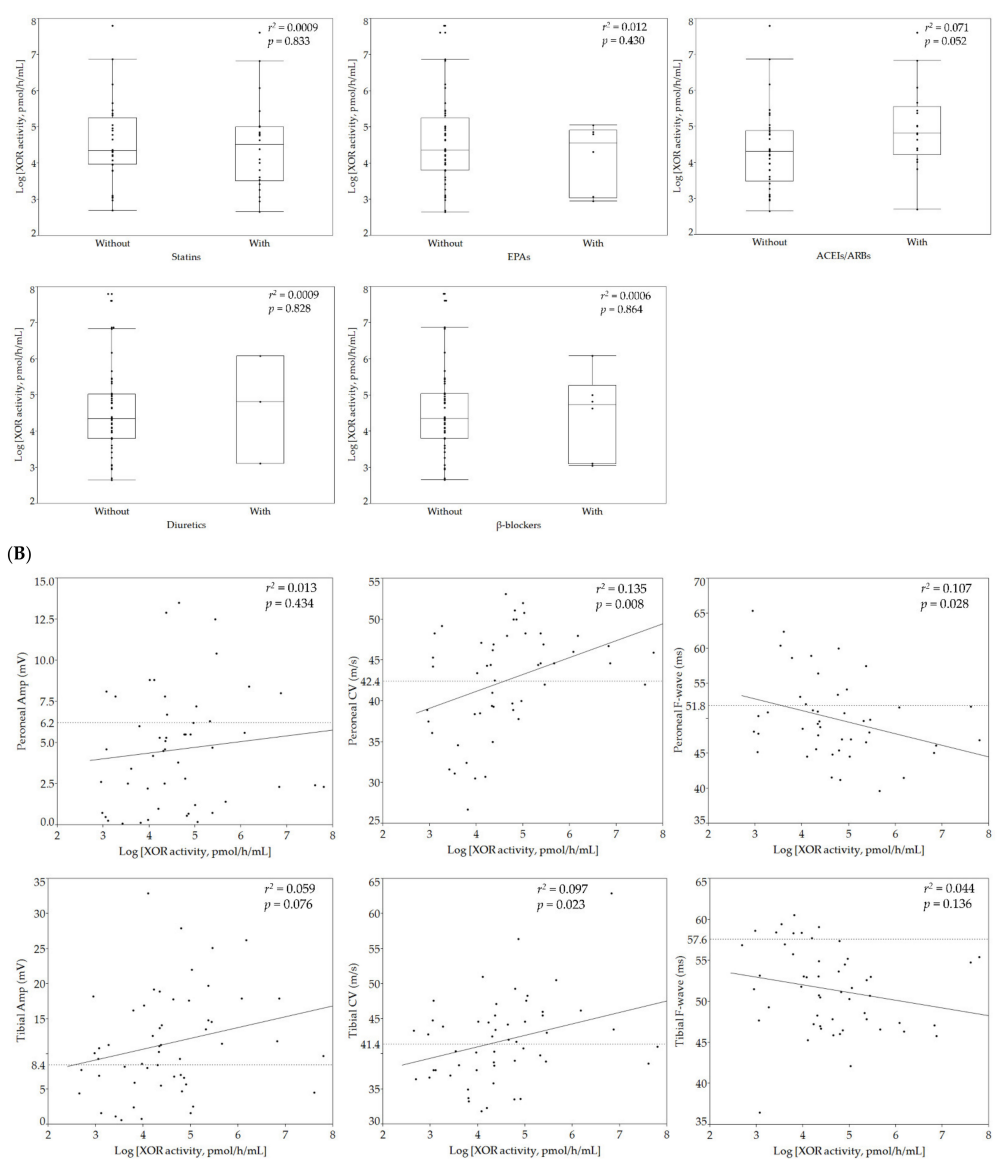
Correlations of individual parameters with logarithmically transformed plasma XOR activity. Results of sex, hypoxanthine, xanthine, UA, age, BMI, waist circumference, VFA, height, SBP, DBP, duration of diabetes, Brinkman index, vibration, minimum ABI, maximum IMT, FBG, HbA1c, Cre, eGFR, ACR, Plt, AST, ALT, γGTP, AAR, APRI, FIB-4 index, Alb, HDL cholesterol, LDL cholesterol, TG, EPA/AA, CRP, BNP, anti-diabetic drugs, antihyperlipidemic agents, and antihypertensive agents are shown in (**A**), those of various NCS parameters in (**B**). Solid lines indicate regression lines, while each dotted line shows the threshold level of each of the NCS parameters in panel (**B**). The thresholds for peroneal/tibial amplitude potential, peroneal/tibial conduction velocity, and peroneal/tibial F-wave for prediction of incident DSP were 6.2/8.4 mV, 42.4/41.4 m/s, and 51.8/57.6 ms, respectively. SBP, systolic blood pressure; DBP, diastolic blood pressure; Cre, serum creatinine; eGFR, estimated glomerular filtration rate; ACR, albumin to creatinine ratio; Plt, platelet count; FIB-4 index, fibrosis-4 index; EPA/AA, eicosapentaenoic acid to arachidonic acid ratio; BNP, B-type natriuretic peptide; CRP, C-reactive protein; αGIs, α-glucosidase inhibitors; GLP-1RAs/DPP4is, GLP-1 agonists/dipeptidyl peptidase-4 inhibitors; SGLT2, sodium-glucose co-transporter 2 inhibitors; ACEIs, angiotensin-converting enzyme inhibitors; ARBs, angiotensin receptor blockers; CCBs, calcium channel blockers; Amp, amplitude potential; CV, conduction velocity; F-wave, F-wave latency. r2: coefficient of determination, vibration: perception time for a fork vibration.

Plasma XOR activity was logarithmically transformed to normalize the skewed distribution to determine correlations between variables using analysis of variance (Figure 1). Correlations of individual parameters with logarithmically transformed plasma XOR activity (ln-XOR) are shown in Figure 2. As shown in panel (A), ln-XOR was significantly and positively correlated with the plasma hypoxanthine level (r2 = 0.086, *p* = 0.032) and the plasma level of xanthine (r2 = 0.439, *p* < 0.0001), but not with the plasma UA level (r2 = 0.123, *p* = 0.011). Ln-XOR showed a significant positive correlation with VFA (r2 = 0.022, *p* = 0.284) but with neither BMI (r2 = 0.024, *p* = 0.265) nor waist circumference (r2 = 0.024, *p* = 0.265). We found that ln-XOR showed a significant negative correlation with the duration of diabetes (r2 = 0.078, *p* = 0.041), the Brinkman index (number of cigarettes smoked per day) x (number of years smoked) (r2 = 0.131, *p* = 0.007), and maximum IMT (r2 = 0.176, *p* = 0.002) but not with sex (r2 = 0.027, *p* = 0.233), age (r2 = 0.014, *p* = 0.400), height (r2 = 0.012, *p* = 0.423), vibration (r2 = 0.012, *p* = 0.443), or minimum ABI (r2 = 0.023, *p* = 0.269). In addition, ln-XOR showed significant positive correlations with aspartate aminotransferase (AST) (r2 = 0.568, *p* < 0.0001), alanine aminotransferase (ALT) (r2 = 0.619, *p* < 0.0001), gamma-glutamyl transferase (γ-GTP) (r2 = 0.188, *p* < 0.0001), the AST to platelet ratio index (APRI) (r2 = 0.310, *p* < 0.0001), and albumin (r2 = 0.093, *p* = 0.025), while showing negative correlations with the AST to ALT ratio (AAR) (r2 = 0.159, *p* = 0.003) and high density lipoprotein (HDL)-cholesterol (r2 = 0.129, *p* = 0.008). The ln-XOR showed no significant correlations with FBG (r2 = 0.153, *p* = 0.372), HbA1c (r2 = 0.0001, *p* = 0.930), estimated glomerular filtration rate (eGFR) (r2 = 0.010, *p* = 0.469), low density lipoprotein (LDL)-cholesterol (r2 = 0.026, *p* = 0.249), triglyceride (TG) (r2 = 0.030, *p* = 0.209), the eicosapentaenoic acid to arachidonic acid ratio (EPA/AA) (r2 = 0.0008, *p* = 0.843), C-reactive protein (CRP) (r2 = 0.006, *p* = 0.571), or B-type natriuretic peptide (BNP) (r2 = 0.066, *p* = 0.061). We examined relationships of XOR with anti-diabetic drugs, antihyperlipidemic agents, and antihypertensive agents and found that ln-XOR showed a significant negative correlation with GLP-1 agonists/dipeptidyl peptidase-4 inhibitors (GLP-1RAs/DPP4is) (r2 = 0.162, *p* = 0.003).

As shown in panel (B), we investigated the correlations between ln-XOR and NCS parameters. For the correlation analysis, we excluded the parameters related to the sural nerve because in half of the analyzed participants (27/54) these parameters were not detectable (Table 1). There were no significant correlations of ln-XOR with peroneal amp (r2 = 0.013, *p* = 0.434), tibial amp (r2 = 0.059, *p* = 0.076), or tibial F-wave (r2 = 0.044, *p* = 0.136). However, we did demonstrate significant positive correlations of ln-XOR with both peroneal conduction velocity (r2 = 0.135, *p* = 0.008) and tibial conduction velocity (r2 = 0.097, *p* = 0.023), together with a significant negative correlation of ln-XOR with the peroneal F-wave (r2 = 0.107, *p* = 0.028).

### 3.3. Relationships of Plasma XOR Activity with NCS Parameters

**Figure 3:**
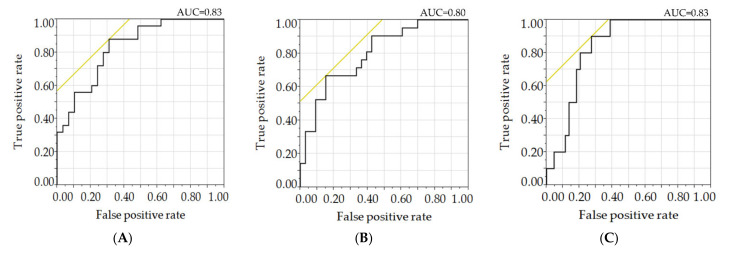
ROC curves for various NCS parameters stratified by the thresholds identified for prediction of incident DSP. (**A**) Peroneal CV, (**B**) peroneal F-wave, (**C**) tibial F-wave. ROC, receiver operating characteristic; AUC, area under the curve; F-wave, F-wave latency. NCV parameters were stratified by the thresholds identified for the prediction of incident DSP according to a previous report as described in the “**Patients and Methods**” section, that is, the thresholds for peroneal conduction velocity, peroneal F-wave, and tibial F-wave were 42.4 m/s, 51.8 ms, and 57.6 ms, respectively.

As for DSP, stepwise multiple regression analysis using various NCS parameters as the objective variable, as well as variables including ln-XOR, sex, age, duration of diabetes, BMI, waist circumference, FBG, minimum ABI, and maximum IMT as explanatory variables, revealed significant correlations of peroneal conduction velocity with ln-XOR, age and waist circumference, peroneal F-wave latency with ln-XOR and BMI, and tibial F-wave latency with ln-XOR when stratified by the thresholds identified for prediction of incident DSP (Table 2). Receiver operating characteristic curves (Figure 3) showed the discriminatory power of the ln-XOR for various NCS parameters, such as peroneal conduction velocity, peroneal F-wave, and tibial F-wave, stratified by the thresholds identified for prediction of incident DSP [31]. Their area under the curve values were 0.83, 0.80, and 0.83, respectively.

**Figure 4:**
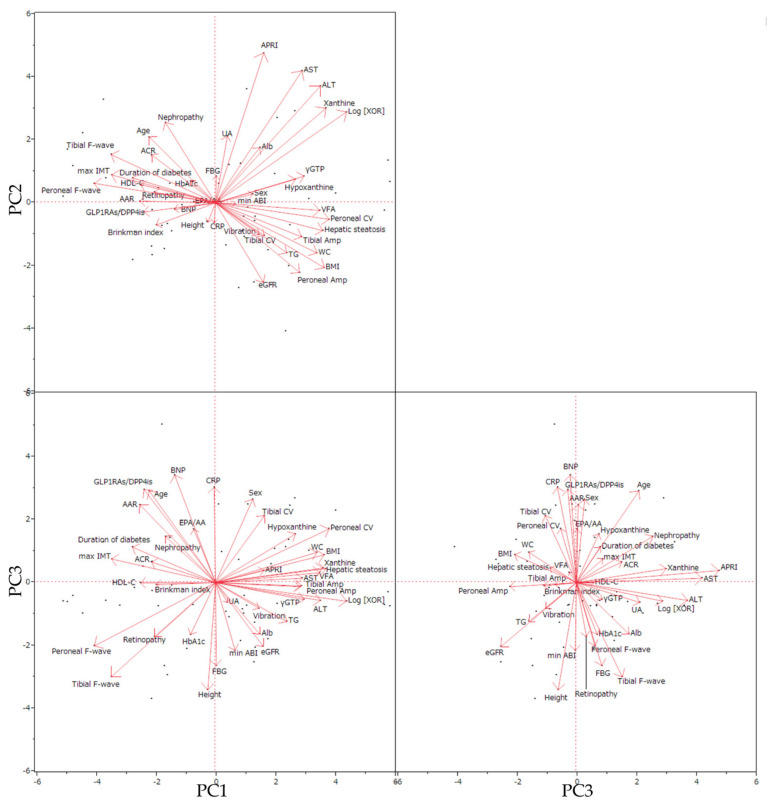
Visualization of correlations between variabilities in PCA biplots. PC, principal component; Amp, amplitude; CV, conduction velocity; WC, waist circumference.

The PCA biplot (Figure 4) showed distributions of parameters from the axes of three primary components (PC). Along the axis of PC1, ln-XOR correlated strongly with hypoxanthine, xanthine, impaired liver functions (ALT, AST, γGTP, and/or APRI abnormalities, with hepatic steatosis), obesity-related parameters (BMI, VFA, waist circumference, and triglyceride), and various NCS parameters. Ln-XOR also correlated positively with amplitude potentials and conduction velocities and negatively with F-waves for both peroneal and tibial nerves.

Factor loadings (Table 3) for PC1 to PC11 showed ln-XOR to have significant loadings in PC1 and PC2. PC1 showed a positive correlation of ln-XOR with impaired liver functions (ALT, AST, γGTP, and/or APRI abnormalities, with hepatic steatosis), xanthine, hypoxanthine, conduction velocity of the peroneal nerve, amplitude potentials (both peroneal and tibial), and obesity-related parameters (BMI, VFA, waist circumference, and triglyceride), as well as negative correlations of ln-XOR with F-waves (both peroneal and tibial), maximum IMT, duration of diabetes, HDL-cholesterol, AAR, GLP1RAs/DPP4is use, retinopathy, ACR, age, and the Brinkman index. PC2 showed positive correlations of ln-XOR with liver dysfunction (APRI, AST, and ALT) and age, while showing inverse relationships of ln-XOR with obesity-related parameters (BMI, waist circumference, and triglyceride), renal function (eGFR and without nephropathy), and peroneal nerve amplitude potential. PC3 showed positive correlations of ln-XOR with F-waves (both peroneal and tibial), height, female gender, FBG, Alb, and minimum AMI, while correlations with age, the AST to ALT ratio, BNP, and CRP together with tibial nerve conduction velocity were negative.

## 4. Discussion

We investigated the associations of plasma XOR activity in T2DM patients with individual parameters and diabetic vascular complications. To our knowledge, this study is the first to demonstrate low levels of plasma XOR activity to be associated with an elongation in the F-wave latencies of both tibial and peroneal nerves, which are known to be the most sensitive parameters of DSP [34]. Stepwise analysis using clinical thresholds for prediction of incident DSP applied for various NCS parameters showed significant and independent sensitivity of XOR activity measurement for the detection of DSP. In contrast, we found that high plasma XOR activity levels were associated with a short duration of diabetes and metabolic disorders often found in young diabetic patients such as central obesity and liver dysfunction.

Recently, relationships between various clinical features and the level of plasma XOR activity measured by the procedure used in our present study have been reported. For example, plasma XOR activity is related to obesity and habitual smoking in the general population [35], vascular endothelial dysfunction assessed by flow-mediated dilation in patients with type 1 diabetes [36], liver dysfunction in T2DM patients [37], the prevalence rate of coronary artery spasm [38], and adverse clinical outcomes in patients with heart failure but with a preserved ejection fraction [39], as well as the requirement for cardiovascular intensive care [40]. Previous studies demonstrated that XOR activity is upregulated in patients with diabetic vascular complications [41,42,43,44]. Furthermore, XOR was suggested to mediate axonal and myelin loss in a murine model of neural diseases [45]. XOR-derived ROS involvement in the pathogenesis of tissue lesions induced by reperfusion after ischemia is thought to be related to vascular injury resulting from diabetic vascular complications [46]. Taken together, these observations suggest that plasma XOR activity is upregulated in the early period of diabetes and then becomes exhausted with the development of diabetic vascular complications, as suggested in a recent report [47].

Moreover, PCA analysis revealed two patterns of relationships between XOR activity and F-wave latency. One pattern is low XOR activity associated with elongation in the F-wave latency, consistent with our main findings, while the other is an inverse weakly positive relationship, possibly reflecting neuronal injury due to altered XOR activity, as shown in previous studies [41,42,43,44]. Importantly, the thresholds for F-wave latency that we used in this study were effective for discriminating DSP due to decreased XOR activity. Interestingly, this study also revealed increases in both incretin and its mimetics, due to the use of GLP1RAs or DPP4is, to be related to low XOR activity, an observation consistent with accumulating evidence that incretins induce anti-inflammatory effects by downregulating ROS production and NF-κB activation in vascular cells [48,49].

Since assessing NCS requires expensive equipment and specialized personnel, in addition to being relatively invasive and time-consuming to perform, the recent position statement from the American Diabetes Association has stressed that diabetic polyneuropathy (DPN) can be clinically diagnosed without assessing NCS results and that NCS is only required in patients with special situations [50]. Diabetologists have been making efforts to establish simpler methods of detecting DPN, but none have been sufficiently reliable to gain worldwide acceptance for diagnosing DPN [51,52,53]. As noted above, the plasma XOR level may serve as a marker for evaluating the development of DPN. Applying this technique to measure plasma XOR levels is rather complicated, based on utilizing a combination of [13C2,15N2]-xanthine and measurement using LC/TQMS. Improvements of this technique are thus needed before a routine clinical application can be achieved.

This study has limitations. First, the collection of blood samples and various physiological examinations were not consistently performed on the same day which might have resulted in bias. Second, there might have been selection bias as our participants were a group of patients willing to be examined for diabetic complications. Furthermore, in order to study patients who had complete NCS data, we analyzed only a portion of the participants in our database because our physiological laboratory incorporated F-wave parameters into the NCS dataset after December 2018. The final study size was thus determined by referring to previous reports, one with 26 [29] and the other with 71 [36] subjects. In our view, reasonable confidence is achieved above a certain standard when a statistically meaningful difference is demonstrated. Third, this study was cross-sectional and observational such that the relationship between diabetic DSP and plasma XOR activity cannot be assumed to be causal. An interventional study with a much larger sample size is needed to further elucidate the association between DSP and plasma XOR activity.

## 5. Conclusions

In this cross-sectional analysis, we showed that plasma XOR activity is a potential predictor of diabetes disease status. XOR activity is upregulated in the early period of diabetes and then appears to become exhausted with the development and progression of diabetic vascular complications. As early DSP usually lacks typical symptoms and is very difficult to detect when employing routine outpatient examinations, measurement of plasma XOR activity might serve as a reliable evaluation tool for DSP prior to the development of symptoms.

## Figures and Tables

**Table 1:** Baseline characteristics of study participants.

Variable (*n* = 54) *	***n* or Mean ± SD**	**(Median)**
Sex (male/female)	37/17	
Age (years)	64.7 ± 12.2	(66.5)
Height (cm)	162.5 ± 9.6	(163.3)
BMI (kg/m^2^)	26.0 ± 5.9	(24.0)
Waist circumference (cm)	94.1 ± 14.8	(93.5)
VFA (cm^2^)	180.8 ± 78.3	(171.4)
SBP (mmHg)	131 ± 19	(130)
DBP (mmHg)	79 ± 13	(78)
Pulse Rate (bpm)	76 ± 12	(77)
Duration of diabetes (M)	146 ± 130	(108)
Brinkman index	443 ± 677	(0)
Vibration (sec)	7.3 ± 3.5	(7.0)
CVRR (%)	148 ± 130	(106)
Minimum ABI	1.07 ± 0.13	(1.11)
Maximum IMT (mm)	2.3 ± 1.2	(2.2)
Laboratory measurements		
XOR activity (pmol/h/mL)	216 ± 441	(78.3)
Hypoxanthine (μM)	1.7 ± 0.8	(1.5)
Xanthine (μM)	0.63 ± 0.30	(0.49)
UA by LC/TQMS (mg/dL)	5.0 ± 1.4	(4.8)
FBG (mg/dL)	168 ± 46	(161)
HbA1c (%)	9.4 ± 1.9	(9.2)
Cre (mg/dL)	0.9 ± 0.4	(0.8)
eGFR (mL/min/1.73 m^2^)	72.9 ± 24.1	(71.1)
ACR (mg/g)	127 ± 288	(30.3)
Plt (×10^9^/L)	223 ± 63	(215)
AST (IU/L)	30 ± 24	(23)
ALT (IU/L)	33 ± 34	(24)
γGTP (IU/L)	54 ± 78	(34)
AAR	1.11 ± 0.61	(1.00)
APRI	0.40 ± 0.37	(0.29)
FIB-4 index	1.92 ± 1.99	(1.50)
Albumin (g/dL)	4.2 ± 0.4	(4.2)
HDL-Chol (mg/dL)	52 ± 15	(49)
LDL-Chol (mg/dL)	122 ± 40	(120)
TG (mg/dL)	136 ± 51	(133)
EPA/AA	0.34 ± 0.31	(0.21)
CRP (mg/dL)	0.3 ± 0.9	(0.1)
BNP (pg/mL)	48.5 ± 28.3	(14.3)
**NCS Parameters**	**□**	**Median (Q1, Q3)**
Sural Amp (μV)		ND (ND, 3.3)
Sural CV (m/s)		ND (ND, 43.3)
Peroneal Amp (mV)		4.6 (1.4, 6.7)
Peroneal CV (m/s)		43.8 (37.7, 47.0)
Peroneal F-wave (ms)		49.2 (45.8, 52.6)
Tibial Amp (mV)		10.6 (6.4, 17.1)
Tibial CV (m/s)		41.4 (37.7, 45.0)
Tibial F-wave (ms)		51.6 (47.3, 55.4)
**Incidence of Diabetic Microangiopathy**		***n***
Nephropathy Stage 1 Stage 2 Stage 3 Stage 4		27 21 4 2
Retinopathy NDR SDR PDR		32 8 14
**Use of Antidiabetic Drugs or Other Medications**		***n* (%)**
Insulins		13 (24)
SU/Glinides		9 (17)
GLP1RAs/DPP4is		27 (50)
SGLT2is		12 (22)
Biguanides		15 (28)
TZDs		2 (4)
αGIs		5 (9)
Epalrestat		1 (2)
Statins		22 (41)
EPAs		6 (11)
ACEI/ARBs		17 (31)
CCBs		15 (28)
Diuretics		3 (6)
β-blockers		6 (11)

SBP, systolic blood pressure; DBP, diastolic blood pressure; Cre, serum creatinine; eGFR, estimated glomerular filtration rate; ACR, albumin to creatinine ratio; Plt, platelet count; FIB-4 index, fibrosis-4 index; EPA/AA, eicosapentaenoic acid to arachidonic acid ratio; CRP, C-reactive protein; BNP, B-type natriuretic peptide; Amp, amplitude potential; ND, not determined; CV, conduction velocity; F-wave, F-wave latency. Vibration: perception time for a fork vibration. F-wave latency was corrected by height as previously described as described in the “**Patients and Methods**” section. All data are presented as *n* or means ± standard deviation and medians with or without the first (Q1) and the third (Q3) quartiles. * Visceral fat area from two patients and vibration data from one patient were missing. The coefficient of variation of RR intervals (CVRR) from eight patients with arrhythmias were omitted. SUs, sulfonylureas; GLP-1RAs/DPP4is, GLP-1 agonists/dipeptidyl peptidase-4 inhibitors; SGLT2: sodium-glucose cotransporter 2 inhibitors; αGIs: α-glucosidase inhibitors; TZDs: thiazolidinediones; EPAs, eicosapentaenoic acids; ACEIs: angiotensin-converting enzyme inhibitors; ARBs: angiotensin receptor blockers; CCBs: calcium channel blockers.

**Table 2:** Univariate and multivariate regression analysis of NCS parameters.

**Univariate Analysis**				
**Objective Variable**	**Explanatory Variable**	**OR**	**<95% CI>**	***p*** **-value**
Peroneal Amp (<6.2 mV)	Ln-XOR	0.78	<0.46–1.31>	0.349
Peroneal CV (<42.4 m/s)		0.48	<0.24–0.83>	0.007
Peroneal F-wave (>51.8 ms)		0.39	<0.18–0.73>	0.002
Tibial Amp (<8.4 mV)		0.63	<0.34–1.07>	0.087
Tibial CV (<41.4 m/s)		0.84	<0.51–1.34>	0.454
Tibial F-wave (>57.6 ms)		0.23	<0.07–0.58>	0.001
**Multivariate Analysis**				
**Objective Variable**	**Explanatory Variable**	**OR**	**<95% CI>**	***p*** **-value**
Peroneal CV (<42.4 m/s)	Ln-XOR	0.47	<0.22–0.86>	0.013
	Age	0.93	<0.87–0.99>	0.027
	Waist circumference	0.92	<0.86–0.96>	0.0004
Peroneal F-wave (>51.8 ms)	Ln-XOR	0.48	<0.21–0.89>	0.017
	BMI	0.83	<0.69–0.96>	0.007
Tibial F-wave (>57.6 ms)	Ln-XOR	0.23	<0.07–0.58>	0.001

OR, odds ratio; CI, confidence interval; Amp, amplitude potential; CV, conduction velocity; F-wave, F-wave latency. NCV parameters were stratified by the thresholds identified for prediction of incident DSP according to a previous report as described in the “**Patients and Methods**” section, that is, the thresholds for peroneal/tibial amplitude potential, peroneal/tibial conduction velocity, and peroneal/tibial F-wave were 6.2/8.4 mV, 42.4/41.4 m/s, and 51.8/57.6 ms, respectively.

**Table 3:** Principal component analysis (PCA).

**Factor Loadings for PC1 to PC11**											
**Variables**	**PC1**	**PC2**	**PC3**	**PC4**	**PC5**	**PC6**	**PC7**	**PC8**	**PC9**	**PC10**	**PC11**
Log [XOR activity, pmol/h/mL]	0.75	0.49									
Peroneal F-wave (ms)	−0.69		−0.34								
Peroneal CV (m/s)	0.65										−0.3
Xanthine (μM)	0.63	0.51									
BMI (kg/m^2^)	0.62	−0.35		0.48							
Hepatic steatosis (Without0/Wth1)	0.61										0.35
ALT (IU/L)	0.6	0.64									
Tibial F-wave (ms)	−0.6		−0.51								
Visceral Fat Area (cm^2^)	0.6			0.52							
max IMT (mm)	−0.6			0.32							
Waist circumference (cm)	0.58			0.52							
γGTP (IU/L)	0.51										
AST (IU/L)	0.49	0.72									
Tibial Amp (mV)	0.49			−0.34					0.38	−0.43	
Peroneal Amp (mV)	0.48	−0.38						0.46			
Duration of diabetes (M)	−0.48					−0.48					0.35
Hypoxanthine (μM)	0.46			0.31				−0.36		−0.35	
AAR (AST/ALT ratio)	−0.43		0.42			−0.39					
HDL-Chol (mg/dL)	−0.43				0.44						
GLP1RAs/DPP4is (Without0/Wth1)	−0.41		0.51						−0.34		
TG (mg/dL)	0.41			0.56							
Age (years)	−0.38	0.36	0.5				0.36				
ACR (mg/g)	−0.37				0.37		−0.41				
Retinopathy (Without0/Wth1)	−0.35			0.46	−0.33	−0.32					
Brinkman index	−0.34					0.43					0.37
APRI (AST to Platelet Ratio Index)		0.82									
Nephropathy (Without0/Wth1)		0.44						0.32			
eGFR (mL/min/1.73 m^2^)		−0.43	−0.35	−0.38	−0.34					0.34	
UA (mg/dL) by LC/TQMS		0.36		0.67				−0.32			
Alb (g/dL)		0.3			0.52		0.35				
BNP (pg/mL)			0.58			0.58					
Height (cm)			−0.58				−0.31				
CRP (mg/dL)			0.52		−0.34	0.64					
Sex (Male0/Female1)			0.45		−0.48			−0.32			
FBG (mg/dL)			−0.45				0.65				
min ABI			−0.37	−0.45	0.49						
Tibial CV (m/s)			0.36					−0.42			
EPA/AA					0.51		0.41			0.41	
HbA1c (%)					−0.51		0.39	0.36			
Vibration (sec)							−0.33		0.5	0.31	
**Eigenvalues of PCs**											
	**Eigenvalues**			**Proportion of Variance**				**Cumulative Proportion**			
PC1	7.63			0.191				0.191			
PC2	3.69			0.092				0.283			
PC3	3.61			0.09				0.373			
PC4	3.22			0.081				0.454			
PC5	2.67			0.067				0.521			
PC6	2.21			0.055				0.576			
PC7	1.81			0.045				0.621			
PC8	1.71			0.043				0.664			
PC9	1.33			0.033				0.697			
PC10	1.26			0.031				0.729			
PC11	1.13			0.028				0.757			

PC, principal component. Hepatic steatosis was diagnosed by ultrasonography. We omitted factor loadings with an absolute value less than 0.3 (indicated as blanks).

## Abbreviations

ABI	Ankle-Brachial Index
AST	Aspartate Aminotransferase
ALT	Alanine Aminotransferase
AAR	AST to ALT Ratio
APRI	AST to Platelet Ratio Index
BNP	B-Type Natriuretic Peptide
BMI	Body Mass Index
CRP	C-Reactive Protein
CVRR	Coefficient of Variation of RR Intervals
DR	Diabetic Retinopathy
DPN	Diabetic Polyneuropathy
DSP	Distal Symmetric Polyneuropathy
EPA/AA	Eicosapentaenoic Acid to Arachidonic Acid Ratio
eGFR	Estimated Glomerular Filtration Rate
FBG	Fasting Blood Glucose
γ-GTP	Gamma-Glutamyl Transferase
HDL-Cholesterol	High Density Lipoprotein-Cholesterol
IMT	Intima-Media Thickness
LC	Liquid Chromatography
ln-XOR	Logarithmically Transformed Plasma XOR Activity
LDL-Cholesterol	Low Density Lipoprotein-Cholesterol
NCS	Nerve Conduction Study
NDR	No Apparent Diabetic Retinopathy
PCA	Principal Component Analysis
PDR	Proliferative Diabetic Retinopathy
SDR	Simple Diabetic Retinopathy
TQMS	Triple Quadrupole Mass Spectrometry
T2DM	Type 2 Diabetes Mellitus
TG	Triglyceride
UA	Uric Acid
VFA	Visceral Fat Area
XOR	Xanthine Oxidoreductase

## References

[1] Rao Kondapally Seshasai S., Kaptoge S., Thompson A., Di Angelantonio E., Gao P., Sarwar N., Whincup P.H., Mukamal K.J., Gillum R.F., Holme I. (2011). Diabetes mellitus, fasting glucose, and risk of cause-specific death. N. Engl. J. Med..

[2] Saeedi P., Petersohn I., Salpea P., Malanda B., Karuranga S., Unwin N., Colagiuri S., Guariguata L., Motala A.A., Ogurtsova K. (2019). Global and regional diabetes prevalence estimates for 2019 and projections for 2030 and 2045: Results from the International Diabetes Federation Diabetes Atlas, 9(th) edition. Diabetes Res. Clin. Pract..

[3] Saeedi P., Salpea P., Karuranga S., Petersohn I., Malanda B., Gregg E.W., Unwin N., Wild S.H., Williams R. (2020). Mortality attributable to diabetes in 20–79 years old adults, 2019 estimates: Results from the International Diabetes Federation Diabetes Atlas, 9th ed. Diabetes Res. Clin. Pract..

[4] Callaghan B.C., Cheng H.T., Stables C.L., Smith A.L., Feldman E.L. (2012). Diabetic neuropathy: Clinical manifestations and current treatments. Lancet Neurol..

[5] Tesfaye S., Boulton A.J., Dyck P.J., Freeman R., Horowitz M., Kempler P., Lauria G., Malik R.A., Spallone V., Vinik A. (2010). Diabetic neuropathies: Update on definitions, diagnostic criteria, estimation of severity, and treatments. Diabetes Care.

[6] Furman D., Campisi J., Verdin E., Carrera-Bastos P., Targ S., Franceschi C., Ferrucci L., Gilroy D.W., Fasano A., Miller G.W. (2019). Chronic inflammation in the etiology of disease across the life span. Nat. Med..

[7] Tesfaye S., Chaturvedi N., Eaton S.E., Ward J.D., Manes C., Ionescu-Tirgoviste C., Witte D.R., Fuller J.H. (2005). Vascular risk factors and diabetic neuropathy. N. Engl. J. Med..

[8] Deng Y., Scherer P.E. (2010). Adipokines as novel biomarkers and regulators of the metabolic syndrome. Ann. NY Acad. Sci..

[9] Facchini F., Chen Y.D., Hollenbeck C.B., Reaven G.M. (1991). Relationship between resistance to insulin-mediated glucose uptake, urinary uric acid clearance, and plasma uric acid concentration. JAMA.

[10] Lin J.D., Chiou W.K., Chang H.Y., Liu F.H., Weng H.F. (2007). Serum uric acid and leptin levels in metabolic syndrome: A quandary over the role of uric acid. Metabolism.

[11] Bjornstad P., Laffel L., Lynch J., El Ghormli L., Weinstock R.S., Tollefsen S.E., Nadeau K.J. (2019). Elevated Serum Uric Acid is Associated with Greater Risk for Hypertension and Diabetic Kidney Diseases in Obese Adolescents with Type 2 Diabetes: An Observational Analysis From the Treatment Options for Type 2 Diabetes in Adolescents and Youth (TODAY) Study. Diabetes Care.

[12] Spatola L., Ferraro P.M., Gambaro G., Badalamenti S., Dauriz M. (2018). Metabolic syndrome and uric acid nephrolithiasis: Insulin resistance in focus. Metabolism.

[13] Zhu D.D., Wang Y.Z., Zou C., She X.P., Zheng Z. (2018). The role of uric acid in the pathogenesis of diabetic retinopathy based on Notch pathway. Biochem. Biophys. Res. Commun..

[14] Yu S., Chen Y., Hou X., Xu D., Che K., Li C., Yan S., Wang Y., Wang B. (2016). Serum Uric Acid Levels and Diabetic Peripheral Neuropathy in Type 2 Diabetes: A Systematic Review and Meta-analysis. Mol. Neurobiol..

[15] Zhang J., Xu C., Zhao Y., Chen Y. (2014). The significance of serum xanthine oxidoreductase in patients with nonalcoholic fatty liver disease. Clin. Lab..

[16] Kuppusamy U.R., Indran M., Rokiah P. (2005). Glycaemic control in relation to xanthine oxidase and antioxidant indices in Malaysian Type 2 diabetes patients. Diabet Med..

[17] Parks D.A., Granger D.N. (1986). Xanthine oxidase: Biochemistry, distribution and physiology. Acta Physiol Scand. Suppl..

[18] Nishino T., Okamoto K., Eger B.T., Pai E.F., Nishino T. (2008). Mammalian xanthine oxidoreductase—Mechanism of transition from xanthine dehydrogenase to xanthine oxidase. FEBS J..

[19] Nishino T., Okamoto K., Kawaguchi Y., Hori H., Matsumura T., Eger B.T., Pai E.F., Nishino T. (2005). Mechanism of the conversion of xanthine dehydrogenase to xanthine oxidase: Identification of the two cysteine disulfide bonds and crystal structure of a non-convertible rat liver xanthine dehydrogenase mutant. J. Biol. Chem..

[20] Gibbings S., Elkins N.D., Fitzgerald H., Tiao J., Weyman M.E., Shibao G., Fini M.A., Wright R.M. (2011). Xanthine oxidoreductase promotes the inflammatory state of mononuclear phagocytes through effects on chemokine expression, peroxisome proliferator-activated receptor-{gamma} sumoylation, and HIF-1{alpha}. J. Biol. Chem..

[21] Kushiyama A., Nakatsu Y., Matsunaga Y., Yamamotoya T., Mori K., Ueda K., Inoue Y., Sakoda H., Fujishiro M., Ono H. (2016). Role of Uric Acid Metabolism-Related Inflammation in the Pathogenesis of Metabolic Syndrome Components Such as Atherosclerosis and Nonalcoholic Steatohepatitis. Mediat. Inflamm..

[22] Murase T., Nampei M., Oka M., Miyachi A., Nakamura T. (2016). A highly sensitive assay of human plasma xanthine oxidoreductase activity using stable isotope-labeled xanthine and LC/TQMS. J. Chromatogr. B Analyt. Technol. Biomed. Life Sci..

[23] Yoshizumi T., Nakamura T., Yamane M., Islam A.H., Menju M., Yamasaki K., Arai T., Kotani K., Funahashi T., Yamashita S. (1999). Abdominal fat: Standardized technique for measurement at CT. Radiology.

[24] Tanaka K., Hara S., Hattori M., Sakai K., Onishi Y., Yoshida Y., Kawazu S., Kushiyama A. (2015). Role of elevated serum uric acid levels at the onset of overt nephropathy in the risk for renal function decline in patients with type 2 diabetes. J. Diabetes Investig..

[25] Haneda M., Utsunomiya K., Koya D., Babazono T., Moriya T., Makino H., Kimura K., Suzuki Y., Wada T., Ogawa S. (2015). A new classification of Diabetic Nephropathy 2014: A report from Joint Committee on Diabetic Nephropathy. Clin. Exp. Nephrol..

[26] Mohamed Q., Gillies M.C., Wong T.Y. (2007). Management of diabetic retinopathy: A systematic review. JAMA.

[27] Ando A., Miyamoto M., Kotani K., Okada K., Nagasaka S., Ishibashi S. (2017). Cardio-Ankle Vascular Index and Indices of Diabetic Polyneuropathy in Patients with Type 2 Diabetes. J. Diabetes Res..

[28] O’Leary D.H., Polak J.F., Wolfson S.K., Bond M.G., Bommer W., Sheth S., Psaty B.M., Sharrett A.R., Manolio T.A. (1991). Use of sonography to evaluate carotid atherosclerosis in the elderly. The Cardiovascular Health Study. CHS Collaborative Research Group. Stroke.

[29] Washio K.W., Kusunoki Y., Murase T., Nakamura T., Osugi K., Ohigashi M., Sukenaga T., Ochi F., Matsuo T., Katsuno T. (2017). Xanthine oxidoreductase activity is correlated with insulin resistance and subclinical inflammation in young humans. Metabolism.

[30] Yanagida H.A.A., Kenta O., Nagasaka S., Ishibashi S., Kotani K., Hasegawa O., Taniguchi N. (2015). Determination of reference ranges for nerve conduction studies: Influence of age, height and gender. Jichi Med. Univ. J..

[31] Weisman A., Bril V., Ngo M., Lovblom L.E., Halpern E.M., Orszag A., Perkins B.A. (2013). Identification and prediction of diabetic sensorimotor polyneuropathy using individual and simple combinations of nerve conduction study parameters. PLoS ONE.

[32] Armstrong T.N., Dale A.M., Al-Lozi M.T., Franzblau A., Evanoff B.A. (2008). Median and ulnar nerve conduction studies at the wrist: Criterion validity of the NC-stat automated device. J. Occup. Environ. Med..

[33] Lee H.J., Kwon H.K., Kim D.H., Pyun S.B. (2013). Nerve conduction studies of median motor nerve and median sensory branches according to the severity of carpal tunnel syndrome. Ann. Rehabil. Med..

[34] Andersen H., Stålberg E., Falck B. (1997). F-wave latency, the most sensitive nerve conduction parameter in patients with diabetes mellitus. Muscle Nerve.

[35] Furuhashi M., Koyama M., Higashiura Y., Murase T., Nakamura T., Matsumoto M., Sakai A., Ohnishi H., Tanaka M., Saitoh S. (2020). Differential regulation of hypoxanthine and xanthine by obesity in a general population. J. Diabetes Investig..

[36] Washio K., Kusunoki Y., Tsunoda T., Osugi K., Ohigashi M., Murase T., Nakamura T., Matsuo T., Konishi K., Katsuno T. (2020). Xanthine oxidoreductase activity correlates with vascular endothelial dysfunction in patients with type 1 diabetes. Acta Diabetol..

[37] Kawachi Y., Fujishima Y., Nishizawa H., Nagao H., Nakamura T., Akari S., Murase T., Taya N., Omori K., Miyake A. (2020). Plasma xanthine oxidoreductase activity in Japanese patients with type 2 diabetes across hospitalized treatment. J. Diabetes Investig..

[38] Watanabe K., Shishido T., Otaki Y., Watanabe T., Sugai T., Toshima T., Takahashi T., Yokoyama M., Kinoshita D., Murase T. (2019). Increased plasma xanthine oxidoreductase activity deteriorates coronary artery spasm. Heart Vessels.

[39] Watanabe K., Watanabe T., Otaki Y., Shishido T., Murase T., Nakamura T., Kato S., Tamura H., Nishiyama S., Takahashi H. (2020). Impact of plasma xanthine oxidoreductase activity in patients with heart failure with preserved ejection fraction. ESC Heart Fail..

[40] Shibata Y., Shirakabe A., Okazaki H., Matsushita M., Goda H., Shigihara S., Asano K., Kiuchi K., Tani K., Murase T. (2020). Plasma xanthine oxidoreductase (XOR) activity in patients who require cardiovascular intensive care. Heart Vessels.

[41] Miric D.J., Kisic B.M., Filipovic-Danic S., Grbic R., Dragojevic I., Miric M.B., Puhalo-Sladoje D. (2016). Xanthine Oxidase Activity in Type 2 Diabetes Mellitus Patients with and without Diabetic Peripheral Neuropathy. J. Diabetes Res..

[42] Liu J., Wang C., Liu F., Lu Y., Cheng J. (2015). Metabonomics revealed xanthine oxidase-induced oxidative stress and inflammation in the pathogenesis of diabetic nephropathy. Anal. Bioanal. Chem..

[43] Xia J., Wang Z., Zhang F. (2014). Association between Related Purine Metabolites and Diabetic Retinopathy in Type 2 Diabetic Patients. Int. J. Endocrinol..

[44] Feoli A.M., Macagnan F.E., Piovesan C.H., Bodanese L.C., Siqueira I.R. (2014). Xanthine oxidase activity is associated with risk factors for cardiovascular disease and inflammatory and oxidative status markers in metabolic syndrome: Effects of a single exercise session. Oxid. Med. Cell Longev..

[45] Honorat J.A., Kinoshita M., Okuno T., Takata K., Koda T., Tada S., Shirakura T., Fujimura H., Mochizuki H., Sakoda S. (2013). Xanthine oxidase mediates axonal and myelin loss in a murine model of multiple sclerosis. PLoS ONE.

[46] Battelli M.G., Bolognesi A., Polito L. (2014). Pathophysiology of circulating xanthine oxidoreductase: New emerging roles for a multi-tasking enzyme. Biochim. Biophys. Acta.

[47] Okuyama T., Shirakawa J., Nakamura T., Murase T., Miyashita D., Inoue R., Kyohara M., Togashi Y., Terauchi Y. (2021). Association of the plasma xanthine oxidoreductase activity with the metabolic parameters and vascular complications in patients with type 2 diabetes. Sci. Rep..

[48] Wang D., Luo P., Wang Y., Li W., Wang C., Sun D., Zhang R., Su T., Ma X., Zeng C. (2013). Glucagon-like peptide-1 protects against cardiac microvascular injury in diabetes via a cAMP/PKA/Rho-dependent mechanism. Diabetes.

[49] Lee Y.S., Park M.S., Choung J.S., Kim S.S., Oh H.H., Choi C.S., Ha S.Y., Kang Y., Kim Y., Jun H.S. (2012). Glucagon-like peptide-1 inhibits adipose tissue macrophage infiltration and inflammation in an obese mouse model of diabetes. Diabetologia.

[50] Pop-Busui R., Boulton A.J., Feldman E.L., Bril V., Freeman R., Malik R.A., Sosenko J.M., Ziegler D. (2017). Diabetic Neuropathy: A Position Statement by the American Diabetes Association. Diabetes Care.

[51] Binns-Hall O., Selvarajah D., Sanger D., Walker J., Scott A., Tesfaye S. (2018). One-stop microvascular screening service: An effective model for the early detection of diabetic peripheral neuropathy and the high-risk foot. Diabet. Med..

[52] Alam U., Jeziorska M., Petropoulos I.N., Asghar O., Fadavi H., Ponirakis G., Marshall A., Tavakoli M., Boulton A.J.M., Efron N. (2017). Diagnostic utility of corneal confocal microscopy and intra-epidermal nerve fibre density in diabetic neuropathy. PLoS ONE.

[53] Selvarajah D., Cash T., Davies J., Sankar A., Rao G., Grieg M., Pallai S., Gandhi R., Wilkinson I.D., Tesfaye S. (2015). SUDOSCAN: A Simple, Rapid, and Objective Method with Potential for Screening for Diabetic Peripheral Neuropathy. PLoS ONE.

